# Contraceptive use among HIV-infected women and men receiving antiretroviral therapy in Lusaka, Zambia: a cross-sectional survey

**DOI:** 10.1186/s12889-016-3070-5

**Published:** 2016-05-12

**Authors:** Nancy L. Hancock, Carla J. Chibwesha, Samuel Bosomprah, Jonathan Newman, Mwangelwa Mubiana-Mbewe, Elizabeth Siyama Sitali, Carolyn Bolton-Moore, Clara Mbwili-Muleya, Benjamin H. Chi

**Affiliations:** Department of Obstetrics and Gynecology, UNC Global Women’s Health, University of North Carolina School of Medicine, 3009 Old Clinic Building, Campus, Box 7577, Chapel Hill, NC 27599-7577 USA; Centre for Infectious Disease Research in Zambia, PO Box 34681, 5032 Great North Road, Lusaka, Zambia; Lusaka District Community Health Office, Ministry of Community Development, Mother and Child Health, PO Box 50827, Lusaka, Zambia

**Keywords:** Family planning, Contraception, HIV infection, HIV positive, Africa, Zambia

## Abstract

**Background:**

Family planning (FP) is an essential health service and an important part of comprehensive HIV care. However, there is limited information about the contraceptive needs of people living with HIV in sub-Saharan Africa, which in turn has hampered efforts to expand and integrate FP services into existing HIV programs.

**Methods:**

We performed a cross-sectional survey to determine FP prevalence and predictors among HIV-positive women and men attending 18 public antiretroviral therapy (ART) clinics in Lusaka, Zambia. Trained peer counselors administered the 10-question survey to those seeking care for five days at each of the target sites.

**Results:**

From February to April 2014, we surveyed 7,046 HIV-infected patients receiving routine HIV services. Use of modern contraception was reported by 69 % of female ART patients and 79 % of male ART patients. However, highly effective contraceptive use and dual method use were low among women (38 and 25 %, respectively) and men (19 and 14 %, respectively). HIV disclosure status (adjusted odds ratio (AOR) = 4.91, 95 % confidence interval (CI) = 3.32–7.24 for women, AOR = 3.58, 95 % CI = 2.39–5.38 for men) and sexual activity in the last 6 months (AOR = 5.80, 95 % CI = 4.51–7.47 for women, AOR = 6.24, 95 % CI = 3.51–11.08 for men) were associated with modern contraceptive use in multivariable regression. Most respondents said they would access FP services if made available within ART clinic.

**Conclusions:**

While FP-ART integration may be a promising strategy for increasing FP service uptake, such services must focus on assessing sexual activity and advocating for dual method use to increase effective contraceptive use and prevent unintended pregnancies.

## Background

Worldwide, 220 million women have an unmet need for contraception and 80 million unintended pregnancies occur yearly [1, 2]. Women living with HIV appear to have a higher rate of unintended pregnancy (51–90 %), compared to broader global estimates (38 %) [3]. In addition, the likelihood of pregnancy for HIV-infected women is up to three times higher following initiation of antiretroviral therapy (ART) [4–6]. Improvements in quality of life and health status; renewed interest in sex and changes in sexual behavior; and high cultural value placed on parenthood likely influence this relationship [7, 8]. However, HIV carries an increased risk of adverse pregnancy outcomes as well, including miscarriage, stillbirth, and mother-to-child HIV transmission [9–11]. Women with HIV are also more likely to die from illness or disease progression during this critical period [10, 12–16]. Helping people infected with HIV achieve their family planning (FP) intentions is an essential preventive health service and has been included as one of the four prongs outlined by the United Nations (UN) to reduce the burden of pediatric AIDS [17].

Integration of FP and HIV services is a cornerstone to achieving global goals for maternal and child health [17–20]. However, current FP-HIV service integration efforts vary widely – from referral-based, co-located clinics to all-inclusive “one provider-one session” approaches. Due to the variety of contextual factors that may influence program outcomes, there is no consensus model for how best to integrate FP-HIV services. Given inherent challenges such as staff turnover, commodity shortages, and inadequate health infrastructure, understanding the use of contraception by ART patients is an initial step toward integration [21, 22]. To inform FP-HIV service integration planning in Lusaka, Zambia, we sought to determine the prevalence of modern contraception and dual method use among ART patients and to identify associated factors.

## Methods

We conducted a cross-sectional survey of FP use among HIV-positive adults receiving HIV treatment in Lusaka, Zambia. We recruited participants from all 18 public sector, government supported ART clinics in the city. Peer counselors from each clinic were trained to administer a short survey in the context of routine care. Because the survey was integrated into routine clinical care, district health providers did not collect detailed information about rates or reasons for non-participation. However, we believe non-response to be essentially random because staff members were instructed to interview everyone as part of their clinic visit. The survey comprised 10 questions designed to determine the patient’s current pregnancy status (or, for male respondents, that of the partner), HIV disclosure status, partner HIV status, contraceptive use including method(s) used, reasons for method non-use, and interest in accessing FP within ART clinics. Patients were also asked if they had been sexually active within the last 6 months and if they wanted a child within the next year. Patients were encouraged to answer questions in relation to their primary partner. All responses were based on self-report; no verification of pregnancy status or contraceptive use was performed. We did not collect data about ART use, though the majority of individuals enrolled in the program have initiated HIV treatment [23]. We conducted the survey over a five-day period in each clinic among consecutively presenting males age 15 or older and females aged 15–49. Five-day sampling intervals were chosen for each facility, out of consideration for operational issues (e.g., staff workload, cost) and the large anticipated sample size. Use of these program assessment data was approved by the University of Zambia Biomedical Research Ethics Committee (Lusaka, Zambia) and the University of North Carolina at Chapel Hill Institutional Review Board (Chapel Hill, NC, USA).

Descriptive statistics were used to determine prevalence of contraceptive use, pregnancy status, future fertility desires, HIV status disclosure, and sexual activity in the last 6 months. Analyses were stratified according to gender of the respondent. Condoms, oral contraceptive pills, injectables, sub-dermal implant, intrauterine device, and sterilization were considered modern contraception; of those, all were considered highly effective except condoms. Dual method use refers to the use of condoms (male or female) concurrently with another method of contraception. These categorizations are based on established definitions in the medical and public health literature [24]. Contraceptive use by male respondents also included contraceptive use by their female partner, if known. For each gender, backward-selection multivariable logistic model was used to identify predictors independently associated with modern contraception and dual method use. Variables were removed if the likelihood ratio *p*-value was greater or equal to 0.2. Standard errors were adjusted to account for intra-site correlation using the ‘cluster’ option in the logistic model. The analysis was performed using Stata 14 MP (Statacorp, College Station, Texas).

## Results

From February to April 2014, 7605 patients from 18 public facilities completed the survey as part of routine care. Of those, responses from 7046 (93 %) were included in the analysis: 447 were excluded due to missing gender and 112 (108 women, 4 men) were excluded because they were outside the target age range. The majority of patients included in the analysis were women (69 %, *n* = 4872). The median age for women was 34 years (interquartile range, IQR 30–40) and for men 40 years (IQR 34–44). Of the 463 participants who were pregnant or had a pregnant partner at the time of the survey, 49 % reported that the pregnancy was unplanned. Overall, 75 % of respondents reported they did not want a child within the next year. Compared to women, men were more likely to report sexual activity in the last 6 months and to report an HIV-positive partner, but less likely to report no partner. Approximately 5 % of women and men had not disclosed their HIV status to their partner. More than 80 % of respondents reported they would access FP services in ART clinic if the services were available (Table 1).

**Table 1:** Characteristics of and reported contraceptive use among HIV-positive respondents receiving antiretroviral therapy (ART) at 18 public sector clinics in Lusaka, Zambia (*N* = 7046)

	Women, *N* = 4872	Men, *N* = 2174
	*n* (%)	*n* (%)
Age	4405	1994
Median (IQR)	34 (30–40)	40 (34–44)
15–24	322 (8)	36 (2)
25–34	1848 (42)	500 (25)
35–49	2225 (51)	1244 (62)
> 49	^a^	218 (11)
Client or partner currently pregnant	4686	1993
Yes	313 (7)	150 (8)
No, don’t know	4373 (93)	1843 (92)
Desire a child in the next year	4173	1839
Yes	1023 (25)	492 (27)
No, don’t know	3150 (75)	1347 (73)
HIV status disclosed to partner	4764	2129
Yes	3706 (78)	1858 (87)
No, don’t know	299 (6)	93 (4)
No partner	759 (16)	178 (8)
Partner’s HIV status	4819	2147
Negative	787 (16)	408 (19)
Positive	2851 (59)	1455 (68)
Unknown	346 (7)	92 (4)
No partner	835 (17)	192 (9)
Sexually active in previous 6 months	4602	2028
Yes	3526 (77)	1718 (85)
Using contraception	4542	2046
Yes	3138 (69)	1608 (79)
No	1404 (31)	438 (21)
Method of contraception used		
Male condom	1867 (60)	1277 (79)
Injectable contraception	484 (15)	124 (8)
Oral contraceptive pill	342 (11)	96 (6)
Sub-dermal implant	252 (8)	59 (4)
Copper IUD	117 (4)	15 (1)
Female condom	23 (1)	23 (1)
Sterilization	11 (0.5)	1
Other	8	7
Dual method use by those using contraception	797 (25)	217 (14)
Reasons for not using family planning^b^		
No partner	639 (46)	134 (31)
Desire pregnancy	250 (18)	71 (16)
Partner opposed	115 (8)	33 (8)
Fear of side effects	101 (7)	12 (3)
Currently pregnant	112 (8)	50 (10)
Other	336 (23)	126 (25)
Would obtain family planning in ART clinic	4140	1941
Yes	3285 (79)	1637 (84)
No, don’t know	855 (21)	304 (16)

*IQR* interquartile range, *IUD* intrauterine device

^a^Women aged > 49 years were excluded

^b^Percentages do not add to 100 as clients could select more than one reason

### Contraceptive use by women

Among the 3138 women using contraception, 99 % reported using modern contraception. Most used only male condoms (60 %). Injectable contraception (15 %) and oral contraceptive pills (11 %) were the next most frequently reported methods used (Table 1). Overall, 38 % of women reported using a highly effective contraceptive method. Among women who reported sexual activity in the last six months but did not want a child within the next year, 36 % reported using a highly effective method. The most common reason for not using contraception was no partner (46 %) and desired pregnancy (18 %). One-quarter of women reported dual method use for pregnancy and sexually transmitted infection prevention. In multivariable analysis (Table 2), women who were older, did not desire a child in the next year, had disclosed their status to their partner, and were sexually active in the last six months had higher odds of using modern contraception. Highly effective contraceptive use was more likely among women who did not desire a child in the next year, had an HIV positive partner, had disclosed their status to their partner, and were sexually active in the last six months (Table 3). Similar trends were observed when we examined associations with dual method use (Table 4).

**Table 2:** Unadjusted and adjusted odds ratio (OR) of characteristics independently associated with current use of modern contraceptive among HIV-positive respondents receiving antiretroviral therapy (ART) at 18 public sector clinic in Lusaka, Zambia (*n* = 4691)

	Women		Men	
	*n* = 3096		*n* = 1595	
Predictors	Unadjusted OR	Adjusted OR^a^	Unadjusted OR	Adjusted OR^a^
	(95 % CI)	(95 % CI)	(95 % CI)	(95 % CI)
Age (years)				
15–24	ref	ref	ref	ref
25–34	1.85 (1.51–2.27)^*^	1.48 (1.16, 1.90)^*^	9.42 (3.84–23.14)^*^	1.67 (0.40–6.96)
35–49	1.49 (1.18–1.86)^*^	1.57 (1.21–2.03)^*^	16.51 (6.99–38.98)^*^	2.70 (0.68–10.73)
> 49			13.77 (5.63–33.67)^*^	2.22 (0.60–8.29)
Desire a child in the next year				
Yes	ref	ref	ref	ref
No/Don’t know	1.14 (0.87–1.49)	2.68 (2.10–3.43)^*^	1.62 (1.23–2.13)^*^	2.77 (1.76–4.37)^*^
HIV status disclosed to partner				
No/don’t know/no partner	ref	ref	ref	ref
Yes	11.81 (8.54–16.34)^*^	4.91 (3.32–7.24)^*^	10.60 (7.39–15.20)^*^	3.58 (2.39–5.38)^*^
Partner’s HIV status				
Negative/Don’t know/No partner	ref	ref	ref	
Positive	4.14 (3.33–5.15)^*^	1.24 (0.99–1.55)	2.71 (1.99–3.69)^*^	
Sexually active in previous 6 months				
No	ref	ref	ref	ref
Yes	9.57 (7.58–12.06)^*^	5.80 (4.51–7.47)^*^	7.35 (5.27–10.26)^*^	6.24 (3.51–11.08)^*^

Modern contraception includes condoms, oral contraceptive pills, injectables, sub-dermal implant, intrauterine device, and sterilization

^*^ *P*-value < 0.01

^a^The estimation method was backward-selection multivariable logistic model (cluster standard error) with removal probability being ≥ 0.2

**Table 3:** Unadjusted and adjusted odds ratio (OR) of characteristics independently associated with current use of highly effective contraception among HIV-positive respondents receiving antiretroviral therapy (ART) at 18 public sector clinic in Lusaka, Zambia (*n* = 4691)

	Women		Men	
	*n* = 1206		*n* = 295	
Predictors	Unadjusted OR	Adjusted OR^a^	Unadjusted OR	Adjusted OR^a^
	(95 % CI)	(95 % CI)	(95 % CI)	(95 % CI)
Age (years)				
15–24	ref	ref	^b^	
25–34	1.25 (0.99–1.57)	0.92 (0.63–1.35)	0.97 (0.72–1.31)	
35–49^c^	0.78 (0.59–1.04)	0.57 (0.38–0.87)	ref	
> 49			0.48 (0.29–0.80)	
Desire a child in the next year				
Yes	ref	ref	ref	ref
No/Don’t know	1.60 (1.25–2.06)^*^	2.28 (1.74–2.99)^*^	2.20 (1.65–2.94)^*^	2.89 (1.99–4.19)^*^
HIV status disclosed to partner				
No/don’t know/no partner	ref	ref	ref	ref
Yes	4.39 (3.32–5.80)^*^	2.32 (1.65–3.26)^*^	7.93 (2.87–21.95)^*^	7.32 (2.12–25.25)^*^
Partner’s HIV status				
Negative/Don’t know/No partner	ref	ref	ref	
Positive	2.36 (2.05–2.71)^*^	1.44 (1.16–1.80)^*^	1.81 (1.33–2.46)	
Sexually active in previous 6 months				
No	ref	ref	ref	ref
Yes	3.19 (2.72–3.74)^*^	2.07 (1.62–2.65)^*^	2.14 (1.45–3.17)^*^	1.60 (0.95–2.70)

Highly effective modern contraception includes oral contraceptive pills, injectables, sub-dermal implant, intrauterine device, and sterilization

^a^The estimation method was backward-selection multivariable logistic model (cluster standard error) with removal probability being ≥ 0.2

^b^No man aged 15–24 years used highly effective modern method

^c^Used as reference for men because 15–24 age category had no man using dual methods, making the age category highly unstable for referent

^*^ *P*-value < 0.01

**Table 4:** Unadjusted and adjusted odds ratios (OR) of characteristics independently associated with dual method use among HIV-positive respondents receiving antiretroviral therapy (ART) at 18 public sector clinic in Lusaka, Zambia (*n* = 1014)

	Women		Men	
	*n* = 797		*n* = 217	
Predictors	Unadjusted OR	Adjusted OR^a^	Unadjusted OR	Adjusted OR^a^
	(95 % CI)	(95 % CI)	(95 % CI)	(95 % CI)
Age (years)				
15–24	ref		^b^	
25–34	1.45 (1.09–1.92)^*^		0.99 (0.70–1.40)	
35–49^c^	0.98 (0.71–1.35)		ref	
> 49			0.54 (0.32–1.40)	
Desire a child in the next year				
Yes	ref	ref	ref	ref
No/Don’t know	1.59 (1.16–2.19)^*^	2.22 (1.66–2.98)^*^	2.16 (1.43–3.26)^*^	4.36 (1.30–4.62)^*^
HIV status disclosed to partner				
No/don’t know/no partner	ref	ref	ref	ref
Yes	8.91 (4.81–16.49)^*^	4.35 (2.61–7.25)^*^	6.74 (2.46–18.46)^*^	2.33 (1.26–4.32)^*^
Partner’s HIV status				
Negative/Don’t know/No partner	ref	ref	ref	
Positive	2.49 (2.09–2.97)^*^	1.34 (1.08–1.67)^*^	1.70 (1.21–2.40)^*^	
Sexually active in previous 6 months				
No	1	1	1	1
Yes	4.88 (3.21–7.42)^*^	2.78 (1.85–4.19)^*^	2.55 (1.40–4.65)^*^	6.24 (3.51–11.08)^*^

Dual method use refers to the use of condoms (male or female) concurrently with another method of contraception

^a^The estimation method was backward-selection multivariable logistic model (cluster standard error) with removal probability being ≥ 0.2

^b^No man aged 15–24 years used highly effective modern method

^c^Used as reference for men because 15–24 age category had no man using dual methods, making the age category highly unstable for referent

^*^ *P*-value < 0.01

### Contraceptive use by men

Of the 1608 men reporting current contraceptive use, 99 % reported using a modern method. Male condoms were most frequently reported (79 %), followed by injectable contraception (8 %) and oral contraceptive pills use (6 %) by the partner (Table 1). Overall, 18 % of men reported use of a highly effective method of contraception. Among men who reported sexual activity in the last six months but did want a child in the next year, 19 % reported using a highly effective method. Similar to women, the most common reason for not using contraception was no partner (31 %) followed by desired pregnancy (16 %). Dual method use was reported by 14 %. In multivariable analysis (Table 2), men who did not want a child in the next year, had disclosed their HIV status to their partner, and were sexually active in the last six months were more likely to use modern contraception. The same variables predicted highly effective contraceptive use and dual method use in multivariable analysis (Tables 3 and 4).

## Discussion

In this large cross-sectional survey in urban Zambia, two-thirds of female and three-quarters of male ART patients reported using modern contraception in their primary relationship. However, dual method use (i.e., concomitant use of hormonal method with condoms) and highly effective contraceptive use (i.e., a modern method other than condoms) were low. Encouragingly, most respondents reported they would access FP services if made available within ART clinic. These findings support calls to improve FP service provision and prevent unintended pregnancies through integrated FP-ART health services.

Compared to an earlier study performed in Lusaka (2009–2010), more women reported hormonal contraceptive use (34 versus 22 %) and dual method use (25 verses 18 %) in our current study [25]. The proportion of HIV positive females using contraception was less than that reported in rural Uganda (69 %) [4], Nigeria (70 %) [26], and South Africa (86 %) [27], but higher than reported in Malawi (46 %) [28], and Zimbabwe (55 %) [29]. One recent study reported contraceptive prevalence of 91 % among sexually active HIV-positive men not desiring pregnancy in the next 6 months in Kenya, Namibia, and Tanzania [30], which is higher than was observed here. While the locations of these different surveys varied (e.g., postpartum clinics, ART clinics), these comparisons highlight the wide variation of contraceptive uptake among people living with HIV in sub-Saharan Africa.

A large proportion of women and men reported using condoms alone for contraception. While condoms are classified as a “modern” method, the rate of pregnancy with typical use is much higher than with other non-barrier contraceptive methods. However, the effectiveness of condoms in preventing sexually transmitted diseases is also important, particularly in settings where HIV is highly prevalent. Concomitant use of condoms alongside more reliable contraceptive methods – i.e., dual method use – has been increasingly emphasized, including by international agencies such as the World Health Organization [24].

Over one-third of patients using hormonal contraception did not report concurrent condom use. Cytochrome P450-inducing ART drugs may reduce hormonal contraceptive effectiveness due to their effect on metabolism, regardless of how the contraceptive is administered [31]. Multiple pharmacokinetic studies and case reports implicate the non-nucleoside reverse transcriptase inhibitor efavirenz in hormonal contraceptive method failure [32–40]. A 12 % failure rate of the levonorgestrel sub-dermal implant was recently reported among women taking an efavirenz-based ART regimen [41]; this is in stark contrast to the typically reported failure rates of <1 % [42–44]. Since the World Health Organization now recommends efavirenz as part of first-line ART, providers should inquire about contraceptive method use at each visit and reinforce dual method use, especially to those using hormonal methods.

In our survey, most ART patients would access FP services if they were integrated within ART clinics. Male condoms are readily available in most public sector HIV care and treatment facilities, but patients who seek additional and/or different methods must go to a separate department for services. For smaller clinics, these services may be located at a different health facility altogether. While FP-ART integration can vary in scope, implementation of integrated models have increased modern contraceptive use and dual method use in Kenya [45], South Africa [46], and Uganda [47]. New efforts in Zambia have focused on making short-term methods (oral contraceptive pills and injectables) available in ART clinics, while strengthening referral networks for long-acting reversible contraception (LARC) such as sub-dermal implants and intrauterine devices. For example, the Zambian Ministry of Health, in partnership with the Society for Family Health, has posted dedicated LARC providers in high-volume public FP clinics to increase coverage of services [48]. In rural settings, such an approach is now being extended via mobile FP clinics to target underserved groups.

The desire for modern contraception among HIV-infected individuals is important but can be difficult to understand in field settings. Such data have obvious benefits for programs, as they can directly inform operational and strategic planning. Understanding the desire for modern contraception, particularly as it relates to pregnancy intentions, may have important implications for efforts to integrate FP and ART services. For example, if a relatively high proportion of individuals desired pregnancy – as has been reported in other studies [49, 50] – there may be a “ceiling” on the uptake of modern contraception, regardless of how efficient service delivery may be. In addition, there is likely heterogeneity across communities and over time. Unfortunately, we did not collect the necessary information to conduct this type of analysis. We recognize this as a limitation of our study and advocate for its inclusion in similar future work.

Our study had other limitations. First, we were unable to investigate certain demographic, socioeconomic, and medical predictors of contraceptive use, such as marital status, education, number of living children, and duration of ART. Unfortunately, these data were not readily available in our program assessment. Second, our external validity may be limited, making it difficult to extend our results to adolescents and young adults. Contraceptive use patterns may vary between the urban setting of Lusaka and rural parts of Zambia. Third, while our inclusion of men was novel – and provides insights from key decision-makers in Zambian households – their knowledge of contraception use by their female partner may be incomplete, affecting the precision of our usage estimates. Fourth, social desirability bias and recall bias may have influenced patient responses to our survey questions, leading to overestimates about key utilization data and preferences [51]. Finally, we present results from a programmatic assessment. While we have a reasonably large sample size, it was not calculated based on *a priori* assumptions about prevalence of FP use. Because of the lack of data from non-respondents, it is also possible that our sample was not fully representative of the clinic population at large.

## Conclusions

Our data suggest that ART patients use condoms for contraception, especially if they are sexually active and have disclosed their HIV status. While condoms are effective for preventing sexually transmitted diseases, pregnancy is much more likely with typical condom use compared to use of other non-barrier contraceptive methods [24]. Thus, targeted interventions are needed to ensure ART patients are aware of contraceptive efficacy and the importance of dual method use, can easily initiate the method that best meets their FP intentions, and have the needed support to continue or switch methods as fertility goals change. Collaborations between reproductive health service providers and ART providers are essential to ensure appropriate integration of FP-HIV care to achieve these objectives. While improvements are underway to increase FP access for people living with HIV, additional research is needed to identify effective integration methods that help reduce unintended pregnancy.

### Ethics and consent to participate

Ethical approval and use of these program assessment data was approved by the University of Zambia Biomedical Research Ethics Committee (Lusaka, Zambia), reference number 007-03-06, and the University of North Carolina at Chapel Hill Institutional Review Board (Chapel Hill, NC, USA), reference number 12–0411.

### Consent to publish

No individual’s details, images, or video are included such that consent to publish is not applicable.

### Availability of data and materials

The data are stored at the Centre for Infectious Disease Research in Zambia (CIDRZ), Lusaka, Zambia. The University of Zambia Biomedical Research Ethics Committee (Lusaka, Zambia) and the Zambian National Health Research Bill 2013 prevent the public publishing of research data, but the information is available upon request.

## Abbreviations

AOR: adjusted odds ratio
ART: anti-retroviral therapy
CI: confidence interval
FP: family planning
IQR: interquartile range
IUD: intrauterine device
LARC: long-acting reversible contraception
UN: United Nations
